# Mechanisms for Catalytic CO Oxidation on SiAu_n_ (*n* = 1–5) Cluster

**DOI:** 10.3390/molecules28041917

**Published:** 2023-02-17

**Authors:** Yang Zhang, Dasen Ren

**Affiliations:** College of Chemical Engineering, Guizhou Minzu University, Guiyang 550025, China

**Keywords:** AuSi, CO, catalytic oxidation, DFT

## Abstract

Significant progress has been made in understanding the reactivity and catalytic activity of gas-phase and loaded gold clusters for CO oxidation. However, little research has focused on mixed silicon/gold clusters (SiAu_n_) for CO oxidation. In the present work, we performed density function theory (DFT) calculations for a SiAu_n_ (*n* = 1–5) cluster at the CAM-B3LYP/aug-cc-pVDZ-PP level and investigated the effects on the reactivity and catalytic activity of the SiAu_n_ cluster for CO oxidation. The calculated results show that the effect is very low for the activation barriers for the formation of OOCO intermediates on SiAu clusters, SiAu_3_ clusters, and SiAu_5_ clusters in the catalytic oxidation of CO and the activation energy barriers for the formation of OCO intermediates on OSiAu_3_, OSiAu_4_, and OSiAu_5_. Our calculations show that, compared with the conventional small Au cluster, the incorporation of Si enhances the catalytic performance towards CO oxidation.

## 1. Introduction

Metal clusters have long been extensively studied due to their unique optical, electronic, and mechanical properties, as well as their wide range of applications in catalysts [1,2,3,4]. Among the various types of metal clusters, Au clusters have attracted much attention as size-tunable prototypes, because they have been shown to have significant catalytic activity [5,6,7,8,9,10,11,12,13,14,15,16,17,18,19]. Chen et al. [20] showed that Au loading and the Au particle size have significant effects on the generation of methanol and CO; with an increase in the Au loading/Au particle size, the generation activity (Au-mass-normalized reaction rate, TOF) for methanol and CO decreases, while the selectivity for methanol generation increases. The decrease in activity is mainly due to the decrease in the dispersion of Au particles, which is the particle size effect. A comprehensive time-resolved Operando-DRIFTS study of the evolution of various surface species during CO oxidation under high-temperature oxidation conditions for Au/CeO_2_ catalysts (Au particle size range: 1.7 ± 0.6~3.7 ± 0.9 nm) was carried out by Huang et al. [13]. A new perspective on size-effect-supported Au catalysts for CO oxidation is presented; different size-dependent reaction pathways contribute to catalytic activity. Gold clusters are easier to aggregate than various other particles because of their higher surface energy [21,22,23]. Experimental and theoretical studies have been carried out to understand the high catalytic activity of gold clusters and particles. Gold clusters exhibit an excellent ability to catalyze a variety of industrially and environmentally important chemical reactions, such as propylene oxidation [24,25,26], the hydrodeoxygenation of guaiacol [27], the catalytic reduction of 4-nitrophenol [28] and graphene oxide [29], and the 1,2-aminoarylation of alkenes with external amines [30]. Luo et al. [4] investigated the conversion of glycerol-free oxidants to the corresponding aldehydes on simply generated small gold clusters using a simple LAL method. The activation mechanisms for C-O, C-H, and O-H bonds were thoroughly investigated to verify the feasibility of an oxidant-free dehydrogenation strategy catalyzed by small gold clusters produced by aqueous-phase synthesis. Azita et al. [9] investigated the methanol generation reaction over two Au/CeO_2_ catalysts with different gold loading by kinetic and in situ spectroscopy (DRIFTS) measurements, using isotopic labeling techniques, to further elucidate the role of the carrier in the methanol generation reaction for CO_2_ and H_2_ over oxidatively loaded gold catalysts. Gold catalysts have emerged as inimitable π-Lewis acids for selective functionalizations of C-C multiple bonds [31]. Patil et al. [32] developed the first process for the gold-catalyzed 1,2-diarylation of alkenes by designing a mechanistic paradigm that integrates ligand-enabled Au(I)/Au(III) catalysis with the intrinsic π-activation ability of gold complexes.

One of the reactions that has received the most attention in the field of heterogeneous catalysis is CO oxidation [33,34,35]. Both experimental and theoretical studies have investigated the catalysis of CO oxidation by cluster [36,37,38,39,40,41,42,43,44,45,46,47]. Payam et al. [36] found that the catalytic activity of Pd/TiO_2_/Ti metal-supported catalysts was improved by the introduction of Zn, especially at Pd:Zn = 2:1, which reduced the binding energy of CO on the surface and improved the dissociative adsorption of oxygen, which facilitated the oxidation of CO. The work of Cai et al. [37] used density flooding theory (DFT) calculations for CO-catalytic oxidation over a single-atom catalyst, Ti/V_2_CO_2_, to investigate the effect of H_2_O on CO-catalytic oxidation performance and its mechanism, and the study revealed the regulatory mechanism of water molecules in the CO oxidation process over Ti/V_2_CO_2_. Chen et al. [38] showed that the presence of oxygen vacancies and active Cu could enhance the oxidation activity of CO on TiO_2_ substrates, as well as a large number of oxygen vacancies, although promoting the generation of O_ads_ also weakened the redox performance of the catalyst. Compared with the effect of oxygen vacancies on the catalytic oxidation performance of CO, Cu^+^ has a stronger effect on the catalytic oxidation performance of CO. Yoshida et al. [39] showed that Au^δ+^ is an adsorption site for CO and that adsorbed water promoted CO oxidation by Au/POM catalysts. This is the first report of CO oxidation using Au/POM catalysts, and their use has the potential to be extended to various gas-phase reactions. Soni et al. [41] used a novel synthesis method for the preparation of Au@Ti-SiO_2_ with an optimum gold NP size (3–5 nm) using the sol–gel method in one step; for Si/Ti ratios of 10 (ATS 10) and 50 (ATS 50), the activity dramatically increased after treatment in a nitrogen flow, with almost 100% conversion of CO at room temperature.

It is generally believed that Au clusters are the most active catalysts for CO oxidation [48,49]. With regards to CO oxidation in gold clusters, over the past few years, experimental [50] and theoretical [51] studies have shown that small gas-phase gold oxide cluster cations with one atom-bound oxygen atom (Au_n_O^+^, *n* = 1–3) are active and selective for the oxidation of CO to CO_2_, which can occur via both Eley–Rideal- and Langmuir–Hinshelwood-like mechanisms. Recently, Zeng and coworkers [52] used ab initio calculations to show that catalytic activity decreases with increasing adsorption amounts. In particular, it has been shown that smaller gold clusters exhibit higher reactivity to CO and O_2_ [16,53]. In addition, the electronic environment in clusters can be tuned by combining foreign atoms [16,53,54,55]. For example, Au–Ag clusters catalyze CO oxidation [56]. GJena et al. revealed the effects of binding single hydrogen atoms in gold clusters through theoretical calculations [57]; this showed that introducing impurities such as H atoms in gold clusters can be highly efficient and cost-effective compared with using pristine gold clusters for CO oxidation. For example, He et al. reported a highly selective Au/ZnO composite catalyst for the one-step oxidative coupling of CO with secondary amines, producing oxamides [58]. SiAu silicides were experimentally reported in early 1964 by Barrow et al. [59]. Pal et al. [60] systematically studied the structural evolution of SiAu_n_ clusters and found that gold clusters have evidently different structures after silicon incorporation. In a previous study, Kiran et al. reported a series of Si–Au clusters [61,62]. Wang et al. showed that they found relatively large embedding energies and small HOMO–LUMO gaps for AuSi_12_ structures, with enhanced chemical activity and good electron transfer properties being revealed [63]. Although the reaction activity of gold clusters strongly depends on their shape and electron distribution [64,65], little is known about the catalytic activity of SiAu_n_ clusters for CO oxidation.

In this work, we explored the effects of the incorporation of Si on the catalytic activity of gold clusters. In order to better study the effects of gold clusters and the incorporation of Si on the catalytic activity of gold clusters, we studied the structure of a SiAu_n_ (*n* = 1–5) cluster and the catalytic oxidation of two CO molecules using it. The activation barriers for these reactions were calculated and compared with those when using the original gold cluster.

## 2. Results and Discussion

The most stable structures of the gold cluster, Au_n_ (*n* = 1–6), as well as the SiAu_n_ (*n* = 1–5) cluster, are provided in Figures S1 and S2 (the figures with the prefix S are provided in the Supplementary Materials). The optimized geometries of the Au_n_ (*n* = 1–6) and SiAu_n_ (*n* = 1–5) clusters are consistent with the structures recently reported in the literature [66,67].

### 2.1. The Interactions of SiAu_n_ (n = 1–5) Cluster with CO and O_2_

**Figure 1:**
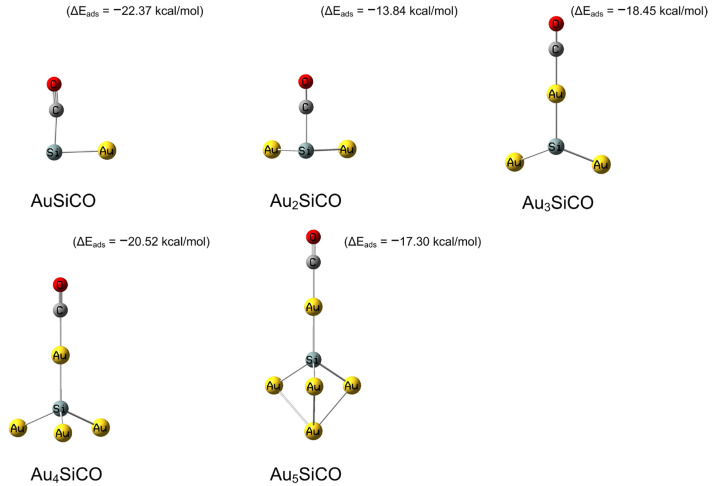
Optimized lowest-energy geometries of CO adsorbed with SiAu_n_ (*n* = 1–5) and the corresponding adsorption energies at the CAM-B3LYP/aug-cc-pVDZ-PP level of theory (in kcal/mol).

The interaction between gold and O_2_ is much weaker than that between gold and CO [15,68]. We first consider the structure and adsorption of carbon monoxide on pristine and SiAu_n_ (*n* = 1–5) clusters, as shown in Figure 1 and Figure S3.

CO adsorption on the gold cluster was repeated to check the reliability of the chosen methods. The favorable adsorption geometries of CO on the Au_n_ (*n* = 1–6) cluster found herein are consistent with the previous results reported in the literature [14,69,70,71]. CO prefers to adsorb at the vertex position of small Au clusters, where top coordination was also found to be preferred. Phala showed that for clusters larger than Au_6_, the top configuration still dominates [72]. Figure S3 shows the adsorption energy of −9.22 kcal/mol for CO on Au, which is in agreement with the result of −9.92 kcal/mol calculated by Tielens et al. [73]. The adsorption energy of CO on Au_2_ is computed to be around −29.75 kcal/mol, which is in agreement with the result of −29.06 kcal/mol calculated by Schwerdtfeger et al. [69]. The adsorption energies of CO on the Au_n_ (*n* = 3–6) cluster are estimated to be −33.44 kcal/mol, −8.30 kcal/mol, −20.52 kcal/mol, and −16.83 kcal/mol, respectively, which are in agreement with the calculations of Wu et al. [14] and Xu et al. [74]. For the Au_n_ (*n* = 1–3) cluster, the CO adsorption energy increases with the increasing cluster size, and for larger clusters of Au_n_ (*n* = 4–6), the CO adsorption energy decreases with the increasing cluster size. This has also been reported in previous investigations [14,70]. This shows that the chosen method is reliable for investigating CO oxidation.

The sites at which CO binds to the SiAu_n_ (*n* = 1–5) cluster exhibit similar features for the Au_n_ (*n* = 1–6) cluster, as listed in Figure 1. The adsorption energy is computed to be −5.07 kcal/mol when CO is adsorbed on Au atoms for SiAu, while it is estimated to be −22.37 kcal/mol for the CO adsorption site on the Si atom of SiAu. When CO is adsorbed on the gold atom of the SiAu_2_ cluster, the adsorption energy is −11.76 kcal/mol, while when it is adsorbed on the Si atom of the SiAu_2_ cluster, the adsorption energy is −13.84 kcal/mol (see Figure S4 for the structure diagram of CO adsorbed on Au atoms). Therefore, for the small SiAu_n_ (*n* = 1–2) cluster, the optimal structure is CO adsorption on the Si site. For the SiAu_n_ (*n* = 3–5) cluster, CO binding to the apex of the SiAu_n_ (*n* = 3–5) cluster is the most stable structure. The adsorption energies of CO on the SiAu_n_ (*n* = 3–5) cluster are −18.45 kcal/mol, −20.52 kcal/mol, and −17.30 kcal/mol, respectively. It is noted that the adsorption energies of CO on the SiAu_n_ (*n* = 1–3) cluster are larger than those of pure golds.

### 2.2. The Oxidation of CO on SiAu_n_ (n = 1–5) Cluster

We consider the catalytic oxidation of CO molecules by SiAu_n_ cluster. Landman and co-workers revealed a Langmuir−Hinshelwood (L-H) type of reaction mechanism for CO oxidation on Au_8_ supported by defect-free and defect-rich magnesia thin films using TPR experiments and ab initio calculations [75]. It is worth noting that transition-metal-bonded gold clusters can act as very efficient catalysts for CO oxidation with quite low activation barriers of 4.61–6.92 kcal/mol [76].

The CO oxidation reaction proceeds via the Langmuir–Hinshelwood (L-H) mechanism or the Eley–Rideal (E-R) mechanism [77], where CO is chemisorbed onto the catalyst and O_2_ is chemisorbed (L-H) or physically adsorbed (E-R) on the catalyst. The first step is the O_2_ reaction with the first CO molecule forming CO_2_ and an adsorbed O atom in Equation (1). The second step is the adsorbed atom O reaction with the second CO molecule to form CO_2_, and this leads to the recovery of the catalyst in Equation (2).

CO + O_2_ + SiAu_n_ = CO_2_ + OSiAu_n_ (1)

OSiAu_n_ + CO = CO_2_ + SiAu_n_ (2)

With regard to even-numbered gold clusters, they are highly active in CO oxidation compared with odd-numbered gold clusters [52,70,78]. In order to compare the effects on CO oxidation with the incorporation of Si on gold clusters, we firstly recalculated the oxidation of CO with a pristine small Au_n_ (*n* = 1–2) cluster (see Figures S5 and S6). The reaction pathway for CO oxidation on Au_2_ is shown in Figure S6. The formation of an OCOO intermediate is not energetically favorable, as the reaction energy is calculated to be 23.75 kcal/mol, as shown in Figure S6.

**Figure 2:**
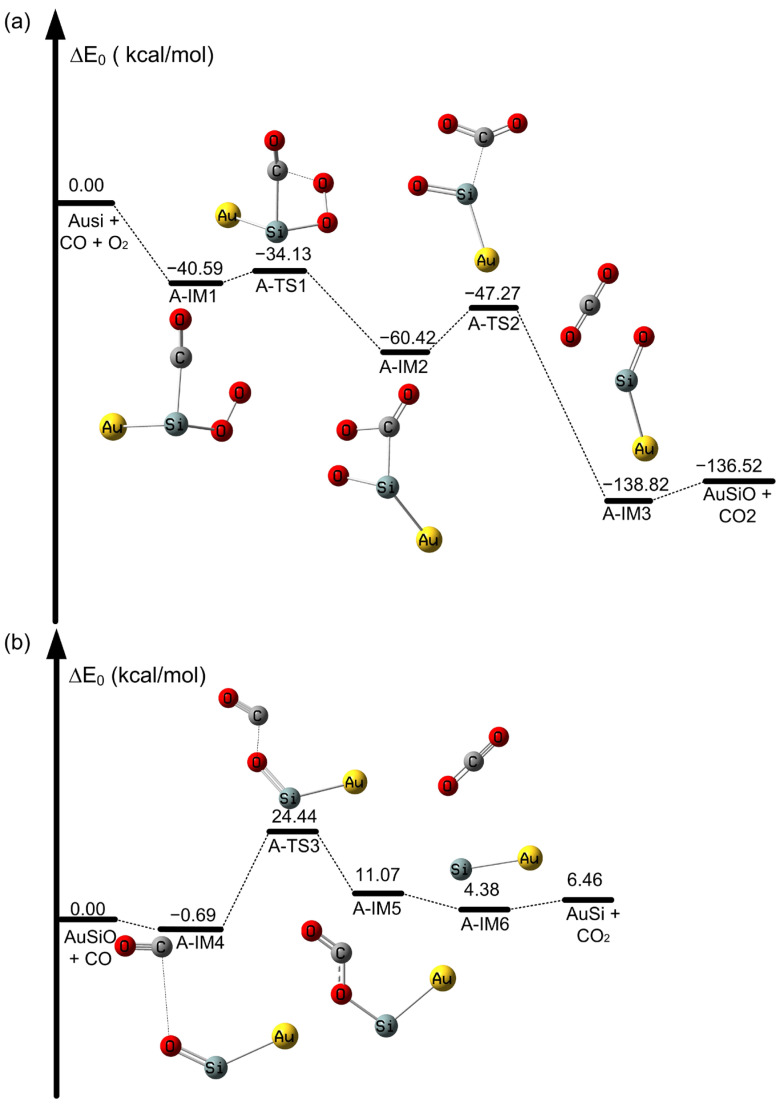
Potential energy profile of CO oxidation on SiAu atoms at the CAM-B3LYP/aug-cc-pVDZ-PP level. (**a**) Oxidation of CO on the SiAu cluster and (**b**) oxidation of CO on the OSiAu cluster (in kcal/mol).

We then considered the oxidation of CO catalyzed by a SiAu cluster, as shown in Figure 2. We note that the rate-determining step for the formation of the OCOO intermediate is from A-IM1 to A-TS1, with an activation barrier of only 6.46 kcal/mol, as depicted in Figure 2a. Moreover, the process is exothermic, with a value of −60.42 kcal/mol. Therefore, the SiAu dimer can remarkably promote the formation of the OCOO intermediate. The formed OCOO intermediate is further decomposed into CO_2_ and OSiAu, with a lower activation barrier of 13.15 kcal/mol from A-IM2 to A-TS2, as listed in Figure 2a. However, from Figure 2b, we find that the activation barrier leading to the formation of OCO intermediate formation from A-IM4 to A-TS3 is very high, with a value of 25.13 kcal/mol on the OSiAu. Moreover, in Figure S6b, the activation barrier for the formation of OCO from G-IM5 to G-TS4 is only 3.23 kcal/mol on the unbonded pristine Au_2_-O cluster. It is noteworthy that the bonded SiAu cluster shows a remarkable improvement in its catalytic effects for the first CO oxidation reaction and the formation of OSiAu compared with the Au_2_ dimer, but the catalytic ability for the second CO is weakened, and the recovery of SiAu is not quite feasible. Thus, the difference in electron transfer from the supports to the Au particles can partially explain the above phenomenon [11].

**Figure 3:**
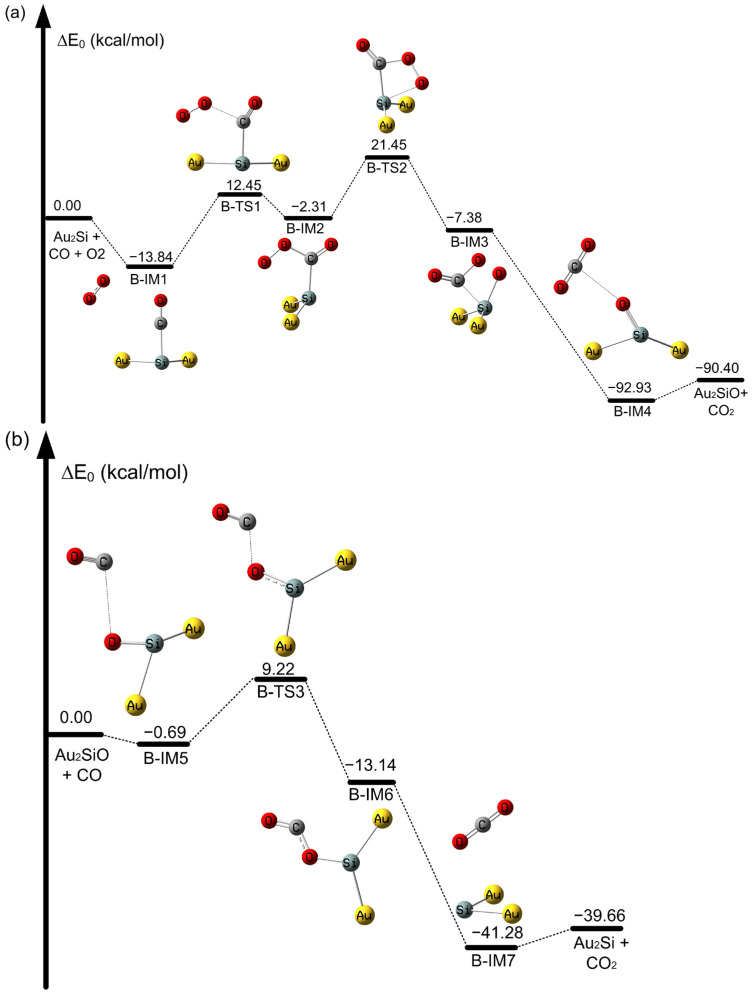
Potential energy profile of CO oxidation on SiAu_2_ atoms at the CAM-B3LYP/aug-cc-pVDZ-PP level. (**a**) Oxidation of CO on the SiAu_2_ cluster and (**b**) oxidation of CO on the OSiAu_2_ cluster (in kcal/mol).

With regard to the Au_3_ cluster, it was found that the rate-determining step of H-IM1 to H-TS1 for the oxidation of CO on Au_3_ to form the OOCO intermediate requires an activation energy barrier of 35.98 kcal/mol, as shown in Figure S7a. In contrast, the activation barrier is only 1.15 kcal/mol for the second CO oxidation process on the Au_3_O cluster, from H-IM5 to H-TS4, to generate OCO intermediates, as listed in Figure S7b. This is in agreement with an earlier investigation [79]. For the oxidation of CO on the SiAu_2_ cluster, the activation energy barrier for the first step from B-IM1 to B-TS1 is 26.29 kcal/mol, leading to the formation of an OOCO intermediate during CO oxidation in Figure 3a. Thus, SiAu_2_ reduces the activation energy barrier for CO oxidation compared with the unbonded pristine Au_3_ cluster. As shown in Figure 3a, the formed OCOO intermediate is decomposed into CO_2_ and OSiAu_2_, with a high activation barrier of 23.76 kcal/mol from B-IM2 to B-TS2. However, from Figure 3b, we find a very high activation barrier for the formation of an OCO intermediate, with a value of 9.91 kcal/mol from B-IM5 to B-TS3 in the case of the Si-bonded OSiAu_2_ cluster. However, on the unbonded pristine Au_3_-O cluster in Figure S7b, the activation barrier for the formation of OCO is only 1.15 kcal/mol. The catalytic ability of the SiAu_2_ cluster is very low for CO oxidation. The slight difference may be attributed to the size effect of gold clusters [80].

**Figure 4:**
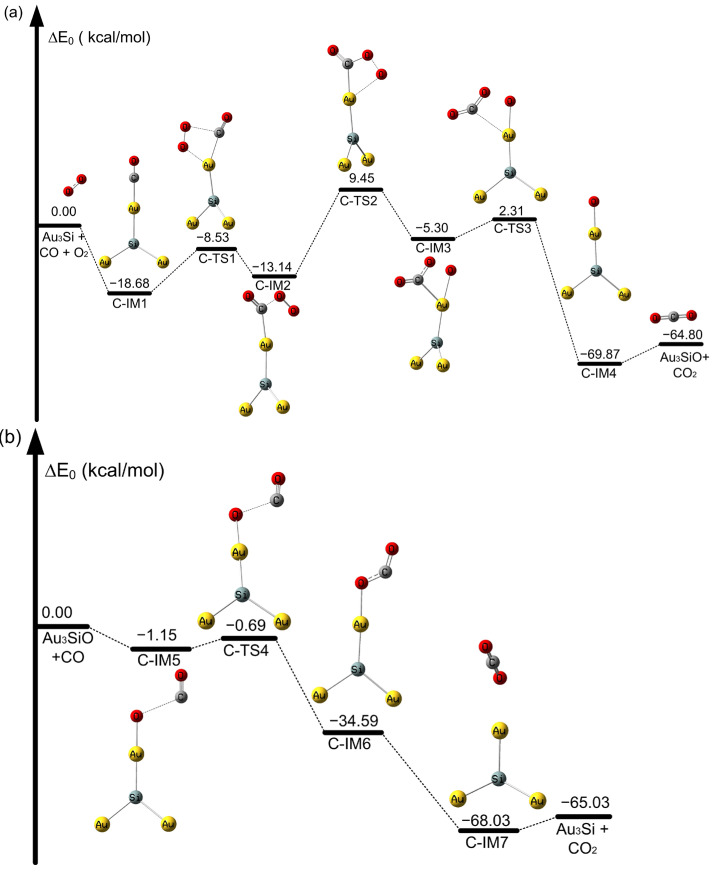
Potential energy profile of CO oxidation on SiAu_3_ atoms at the CAM-B3LYP/aug-cc-pVDZ-PP level. (**a**) Oxidation of CO on the SiAu_3_ cluster and (**b**) oxidation of CO on the OSiAu_3_ cluster (in kcal/mol).

The oxidation of CO in a naked Au_4_ cluster occurs through I-IM1 to I-TS1, with a high activation barrier of 37.82 kcal/mol to form OCOO intermediates, as listed in Figure S8a. This high activation barrier was observed in an earlier investigation [56]. From Figure 4a, we note that for the first CO oxidation on SiAu_3_, the activation barrier is only 10.15 kcal/mol from C-IM1 to C-TS1 to form the OOCO intermediate. Moreover, the process is exothermic, with a value of −13.14 kcal/mol. Therefore, the SiAu_3_ dimer can significantly contribute to the formation of OOCO intermediates. As shown in Figure 4a, the formed OCOO intermediate is decomposed into CO_2_ and OSiAu_3_, with a high activation barrier of 22.59 kcal/mol from C-IM2 to C-TS2. From Figure 4b, we observe that, in the case of Si incorporation into the SiAu_3_-O cluster, the reaction occurs by C-IM5 to C-TS3, with a very low activation barrier of 0.46 kcal/mol for the formation of an OCO intermediate. However, the activation barrier for OCO formation from I-IM3 to I-TS2 is 2.54 kcal/mol in the case of unbonded pristine Au_4_-O in the cluster of Figure S8b. Thus, although the bonded SiAu_3_ cluster reduces the activation energy required for CO oxidation compared with the pristine Au_4_, the decomposition of the OCOO intermediate on SiAu_3_ still needs to overcome a high activation barrier. The reason for this phenomenon may be due to the different active sites on the Au_4_ and SiAu_3_ clusters, which affect catalytic ability.

**Figure 5:**
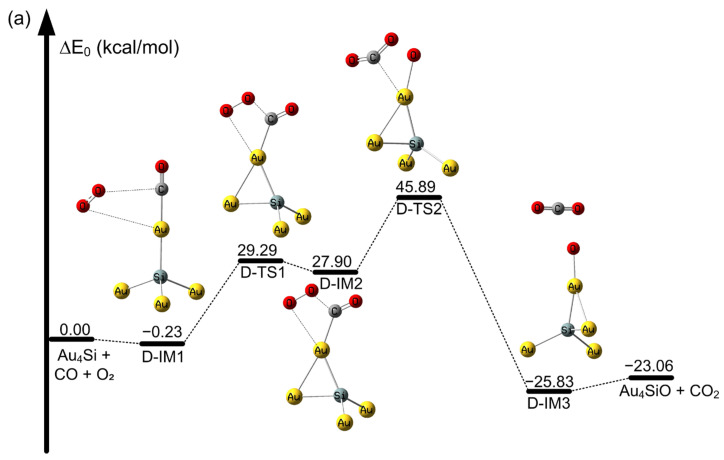

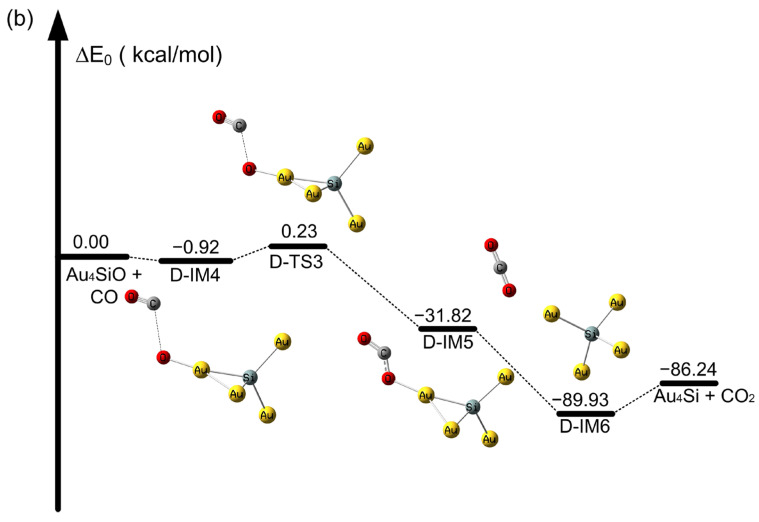
Potential energy profile of CO oxidation on SiAu_4_ atoms at the CAM-B3LYP/aug-cc-pVDZ-PP level. (**a**) Oxidation of CO on the Au_4_Si cluster and (**b**) oxidation of CO on the OSiAu_4_ cluster (in kcal/mol).

With regard to CO oxidation on the Au_5_ cluster, the CO oxidation on the Au_5_ cluster requires an activation energy barrier of 12.45 kcal/mol via J-IM1 to J-TS1 to form the OOCO intermediate, as listed in Figure S9a, which is consistent with the reported values of 13.84 kcal/mol and 18.45 kcal/mol, as calculated by Hyesung et al. in the literature [81]. From Figure 5a, we find that the first CO oxidation process on SiAu_4_ passes through D-IM1 to D-TS1, thus overcoming an activation energy barrier of 29.52 kcal/mol to form the OOCO intermediate. In addition, the process is endothermic, with a value of 27.90 kcal/mol. Moreover, as shown in Figure 5a, passing through D-IM2 to D-TS2, the formed OCOO intermediate is decomposed into CO_2_ and OSiAu_4_, with an activation barrier of 17.99 kcal/mol; this shows that the catalytic ability of SiAu_4_ is much weaker compared with the Au_5_ cluster. From Figure 5b, we find that for the oxidation process of the second CO on OSiAu_4_, only a very low 1.15 kcal/mol energy barrier needs to be passed from D-IM4 to D-TS3 for the formation of the OCO intermediate. As for the oxidation process of CO on Au_5_O, an activation energy barrier of 11.30 kcal/mol is required to form the OCO intermediate, which is consistent with the calculation of 10.38 kcal/mol by Liu et al. [68]. Therefore, the second CO oxidation OSiAu_5_ is very feasible compared with the OAu_5_ cluster. These differences may be due to different active sites and electronic structures [82].

**Figure 6:**
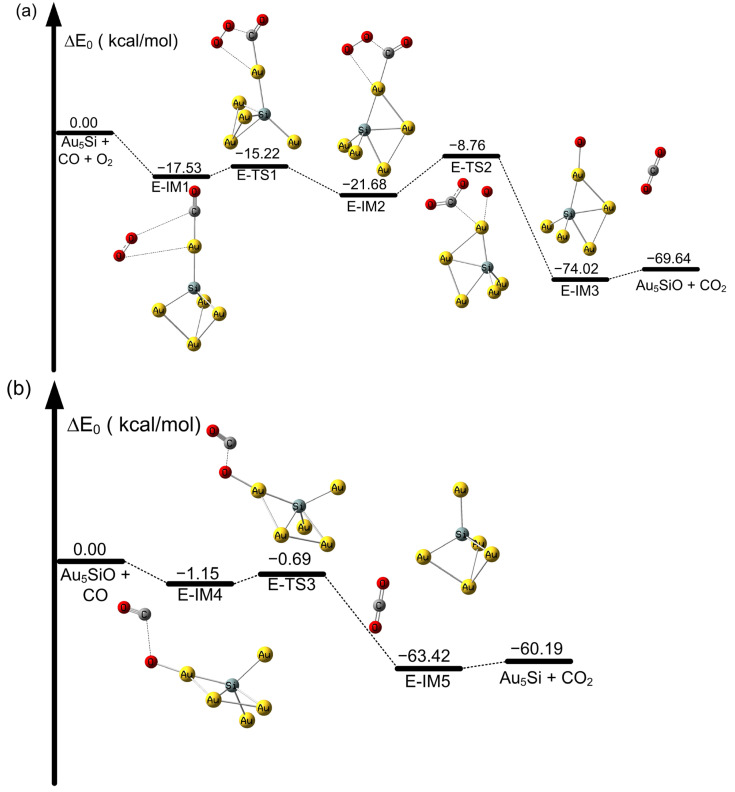
Potential energy profile of CO oxidation on SiAu_5_ atoms at the CAM-B3LYP/aug-cc-pVDZ-PP level. (**a**) Oxidation of CO on the Au_5_Si cluster and (**b**) oxidation of CO on the OSiAu_5_ cluster (in kcal/mol).

With regard to the Au_6_ cluster, as shown in Figure S10a, the oxidation process of CO on the Au_6_ cluster requires a very high activation energy barrier of 32.29 kcal/mol from K-IM1 to K-TS1 for the formation of OOCO intermediates. This high activation barrier has been observed in previous investigations by Ramesh et al. [83]. As shown in Figure 6a, an activation energy barrier of 2.31 kcal/mol is required to form the OOCO intermediate from E-IM1 to E-TS1 during the first CO oxidation on SiAu_5_. Thus, the SiAu_5_ dimer can significantly contribute to the formation of OCOO intermediates. In addition, the process is exothermic, with a value of −21.68 kcal/mol. From Figure 6a, we note that after E-IM2 to E-TS2, the formed OCOO intermediate is then decomposed into CO_2_ and OSiAu_5_, with a relatively low activation energy barrier of 12.92 kcal/mol. The same effect is also shown for the second CO oxidation process. From Figure S10b, we find that the oxidation process of CO on Au_6_O requires a 2.07 kcal/mol activation energy barrier from K-IM5 to K-TS3 for producing the OCO intermediate. However, as shown in Figure 6b, on the OSiAu_5_ cluster, only a very low 0.46 kcal/mol activation energy barrier is required to pass through E-IM4 to E-TS3 for the formation of OCO intermediates. The structure is changed before and after binding, and the charge is transferred. Thus, the bound SiAu_5_ cluster lowers the activation energy barrier for CO oxidation, and the reaction occurs more readily compared with the Au_6_ cluster process. In addition, we also noted that the reactivity and catalytical effects are also affected by supports for different metal oxide surfaces [5,9,20]. Moreover, experimental investigations are important for understanding CO oxidation on SiAu_n_ clusters. We will consider these investigations in the next step of our research.

## 3. Computational Methods

To determine the most stable structures for the SiAu_n_ (*n* = 1–5) cluster, we used the artificial bee colony (ABC) algorithm to carry out a global search by using the ABCluster program [84,85]. Initially, we used the ABCluster program to generate 100 cluster structures for SiAu_n_ (*n* = 1–5) by the B3LYP functional [86]. The aug-cc-pVDZ basis [87,88,89,90,91] set was selected for Si atoms. Pseudopotential ECP60MDF and aug-cc-pVDZ-PP basis [89,90] sets were used for Au atoms. After completing the structure search, we picked out the lowest-energy structures and reoptimized them again by using the CAM-B3LYP functional [91], a long-range-corrected hybrid GGA functional.

We used the CAM-B3LYP functional to perform geometry optimizations and frequency calculations of the stationary points for CO oxidation on the SiAu_n_ cluster. The aug-cc-pVDZ basis set was selected for C, Si, and O atoms. Pseudopotential ECP60MDF and aug-cc-pVDZ-PP basis sets were used for Au atoms. The calculated results indicate that none of the stationary points have imaginary frequencies, except for the transition state, which has only one imaginary frequency. Intrinsic reaction coordinate calculations [92,93,94,95] were performed by using the HPC algorithm to determine the transition states connected with the corresponding reactants and products. In addition, the stability of the DFT wave functions of all species was tested by using the Gaussian 16 keyword (stable = opt) [96,97]. All of the DFT calculations were performed by using the Gaussian 16 software package [98].

## 4. Conclusions

In conclusion, the calculated results show the effects of CO oxidation on the reaction activity and catalytic activity of silicon-bound gold clusters. The results show that compared with the original small Au_n_ (*n* = 1–3) cluster, the clusters incorporating silicon SiAu_n_ (*n* = 1–2) have higher catalytic activity for the first CO oxidation reaction and a lower activation barrier, while the catalytic effects of the second CO oxidation reaction did not improve. For the SiAu_n_ (*n* = 3 or 5) cluster, we found that compared with the Au_n_ (*n* = 4 or 6) cluster, the activation energy barriers were reduced for both the first CO oxidation reaction and the second CO oxidation reaction. In addition, we found that the catalytic performance of incorporating silicon atoms on an odd Au cluster is better than that of an even Au cluster. To sum up, our results emphasize the importance of incorporating heterogeneous impurities in the design of gold clusters with catalytic activity.

## Data Availability

Not applicable.

## Supplementary Materials

The following supporting information can be downloaded at: https://www.mdpi.com/article/10.3390/molecules28041917/s1, Figure S1. Geometrical optimizations of the lowest-energy structures of Au_n_ (*n* = 1–6) based on theory at the CAM-B3LYP/aug-cc-pVDZ-PP level. Figure S2. Geometrical optimizations of the lowest-energy structures of Au_n_Si (*n* = 1–5) based on theory at the CAM-B3LYP/aug-cc-pVDZ-PP level. Figure S3. Optimized lowest-energy geometries of CO adsorbed with Au_n_ (*n* = 1–6) and the corresponding adsorption energies at the CAM-B3LYP/aug-cc-pVDZ-PP level (in kcal/mol). Figure S4. CO adsorption on Au atoms of Au_n_ (*n* = 1–2) cluster structures and the corresponding adsorption energies at the CAM-B3LYP/aug-cc-pVDZ-PP level (in kcal/mol). Figure S5. Potential energy profile of CO oxidation on Au atoms at the CAM-B3LYP/aug-cc-pVDZ-PP level. (a) Oxidation of CO on the Au cluster and (b) oxidation of CO on the OAu cluster (in kcal/mol). Figure S6. Potential energy profile of CO oxidation on Au_2_ atoms at the CAM-B3LYP/aug-cc-pVDZ-PP level. (a) Oxidation of CO on the Au_2_ cluster and (b) oxidation of CO on the OAu_2_ cluster (in kcal/mol). Figure S7. Potential energy profile of CO oxidation on Au_3_ atoms at the CAM-B3LYP/aug-cc-pVDZ-PP level. (a) Oxidation of CO on the Au_3_ cluster and (b) oxidation of CO on the OAu_3_ cluster (in kcal/mol). Figure S8. Potential energy profile of CO oxidation on Au_4_ atoms at the CAM-B3LYP/aug-cc-pVDZ-PP level. (a) Oxidation of CO on the Au_4_ cluster and (b) oxidation of CO on the OAu_4_ cluster (in kcal/mol). Figure S9. Potential energy profile of CO oxidation on Au_5_ atoms at the CAM-B3LYP/aug-cc-pVDZ-PP level. (a) Oxidation of CO on the Au_5_ cluster and (b) oxidation of CO on the OAu_5_ cluster (in kcal/mol). Figure S10. Potential energy profile of CO oxidation on Au_6_ atoms at the CAM-B3LYP/aug-cc-pVDZ-PP level. (a) Oxidation of CO on the Au_6_ cluster and (b) oxidation of CO on the OAu_6_ cluster (in kcal/mol). Figure S11. The structure and energy of various substances in the oxidation pathway for CO on AuSi (in kcal/mol). Figure S12. The structure and energy of various substances in the oxidation pathway for CO on Au_2_Si (in kcal/mol). Figure S13. The structure and energy of various substances in the oxidation pathway for CO on Au_3_Si (in kcal/mol). Figure S14. The structure and energy of various substances in the oxidation pathway for CO on Au_4_Si (in kcal/mol). Figure S15. The structure and energy of various substances in the oxidation pathway for CO on Au_5_Si (in kcal/mol).
