# Supplementary material for: Mechanisms for Catalytic CO Oxidation on SiAun (n = 1–5) Cluster
Source: Molecules. 2023 Feb 17;28(4):1917. doi: 10.3390/molecules28041917 (PMC9962203; doi:10.3390/molecules28041917)
Supplement: Supplementary file 1 [file molecules-28-01917-s001.zip › molecules-2149074-supplementary.pdf]

Supporting Information

Mechanisms for Catalytic CO Oxidation on $SiAu_n$ (n = 1–5) Cluster

Yang Zhang and Dasen Ren *

College of Chemical Engineering, Guizhou Minzu University, Guiyang 550025, China
* Correspondence: dsren@gzmu.edu.cn

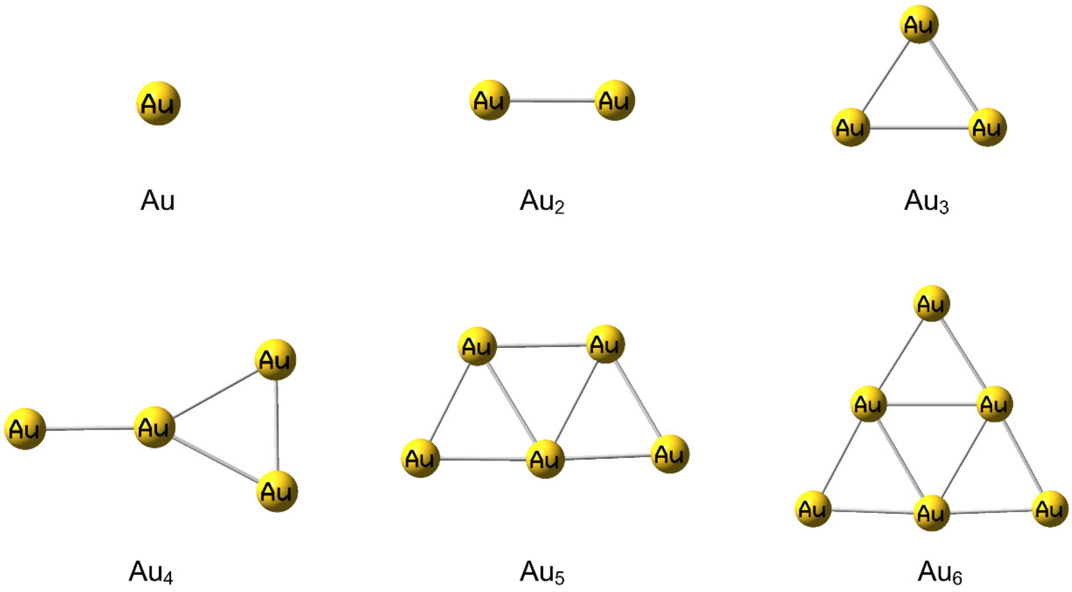

Figure S1. Geometrical optimizations of the lowest-energy structures of Au_n (n=1-6) based on theory at the CAM-B3LYP/aug-cc-pVDZ-PP level.

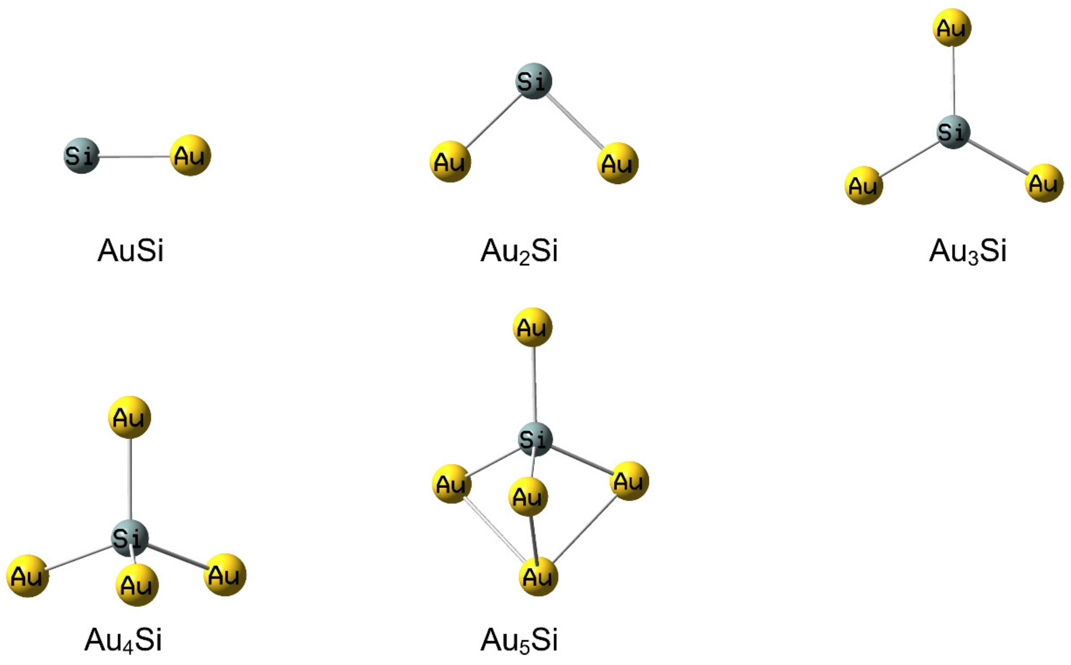

Figure S2. Geometrical optimizations of the lowest-energy structures of Au_nSi (n=1-5) based on theory at the CAM-B3LYP/aug-cc-pVDZ-PP level.

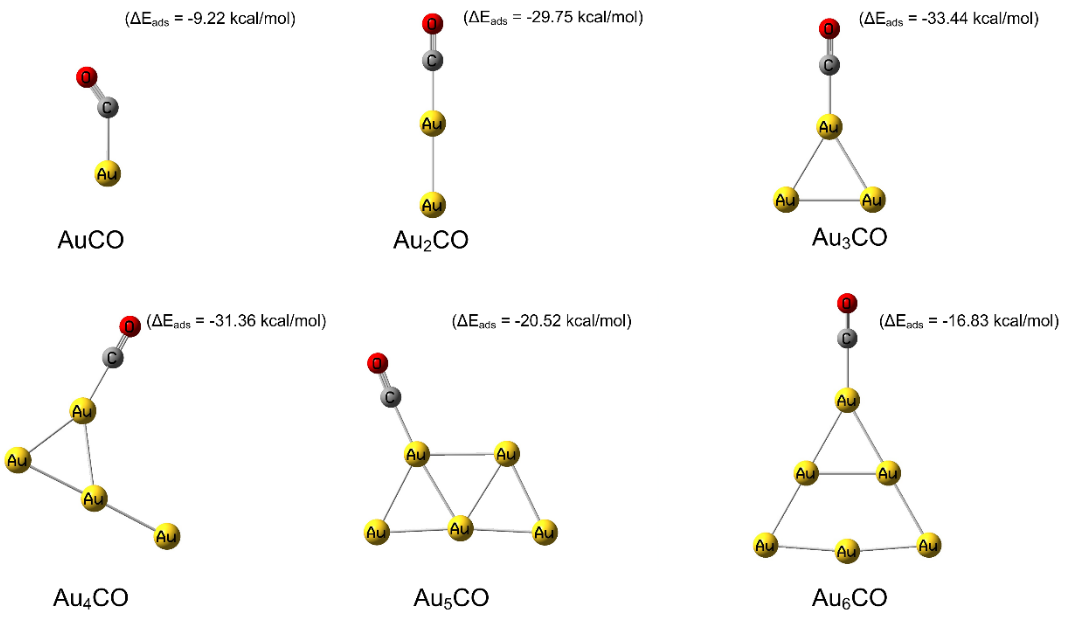

Figure S3. Optimized lowest-energy geometries of CO adsorbed with Au_n (n = 1-6) and the corresponding adsorption energies at the CAM-B3LYP/aug-cc-pVDZ-PP level of theory (in kcal/mol).

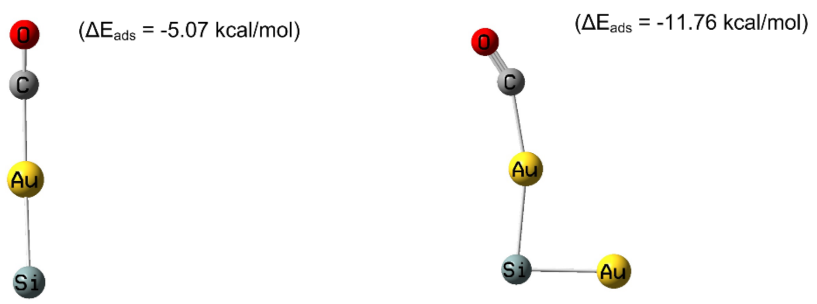

AuSiCO Au$_2$SiCO

Figure S4. CO adsorption on Au atoms of Au$_n$ (n = 1-2) cluster structures and the corresponding adsorption energies at the CAM-B3LYP/aug-cc-pVDZ-PP level of theory (in kcal/mol).

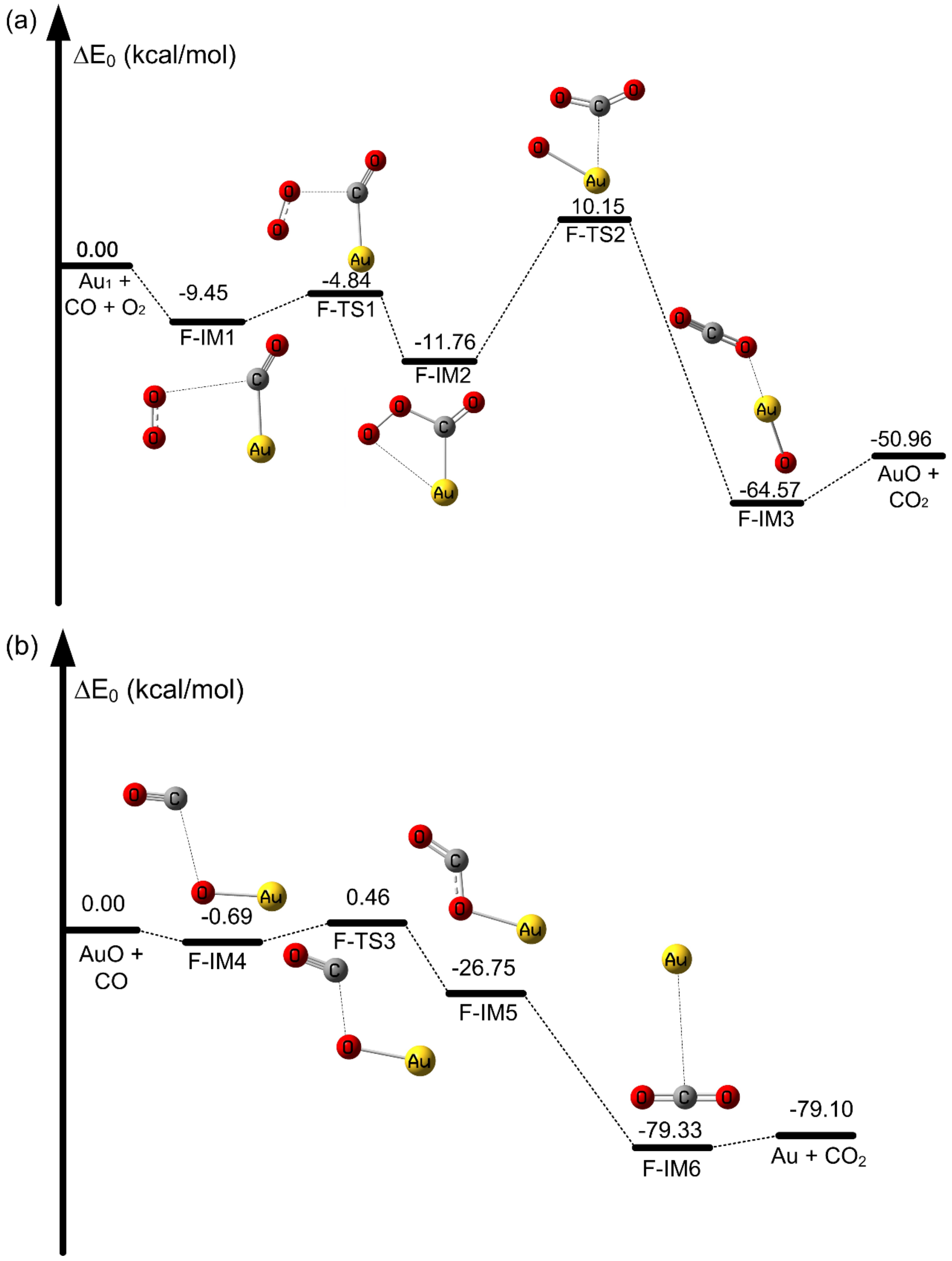

Figure S5. Potential energy profile of CO oxidation on Au atoms at the CAM-B3LYP/aug-cc-pVDZ-PP level. (a) Oxidation of CO on the Au cluster and (b) oxidation of CO on the OAu cluster (in kcal/mol).

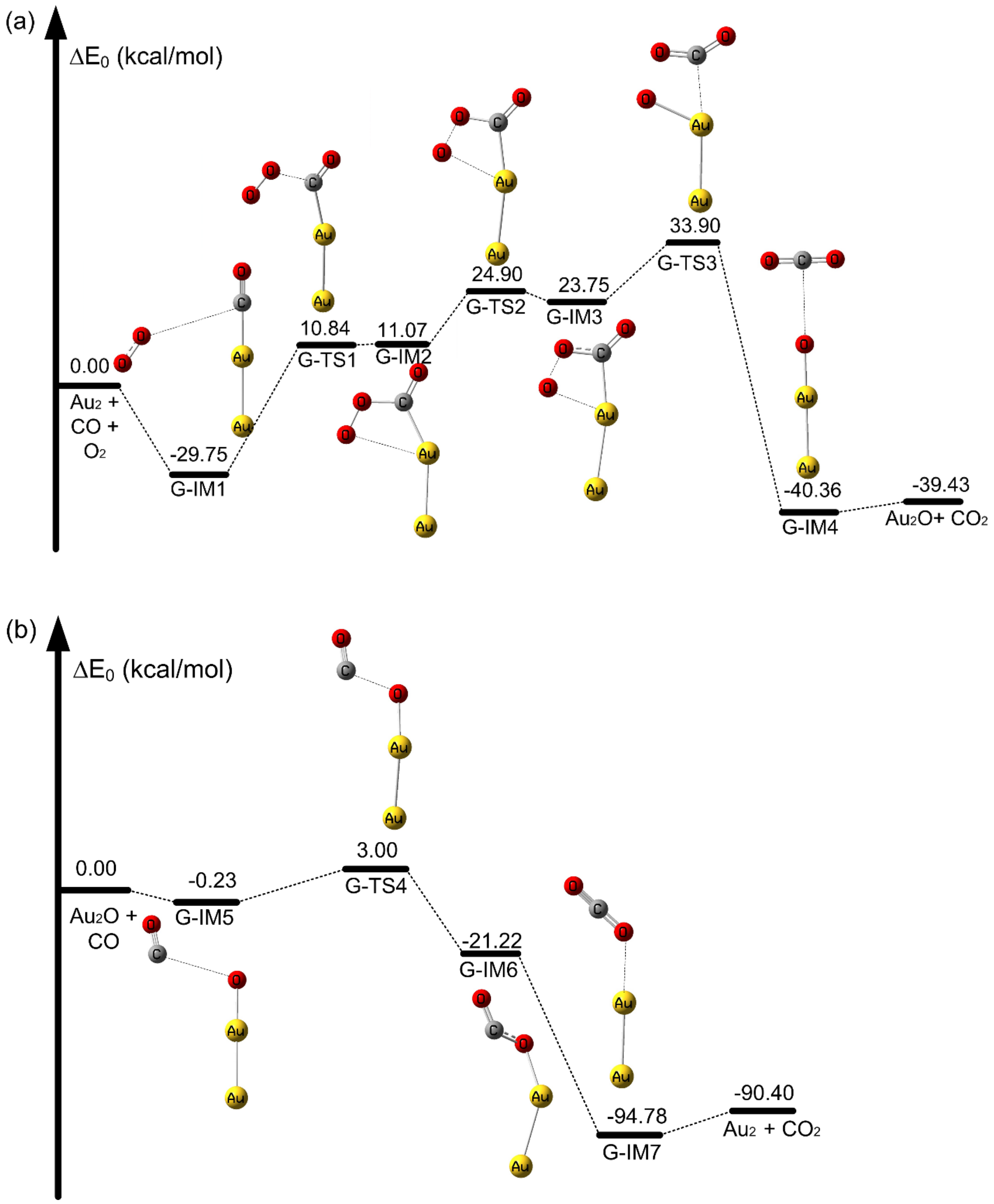

Figure S6. Potential energy profile of CO oxidation on Au_2 atoms at the CAM-B3LYP/aug-cc-pVDZ-PP level. (a) Oxidation of CO on the Au_2 cluster and (b) oxidation of CO on the OAu_2 cluster (in kcal/mol).

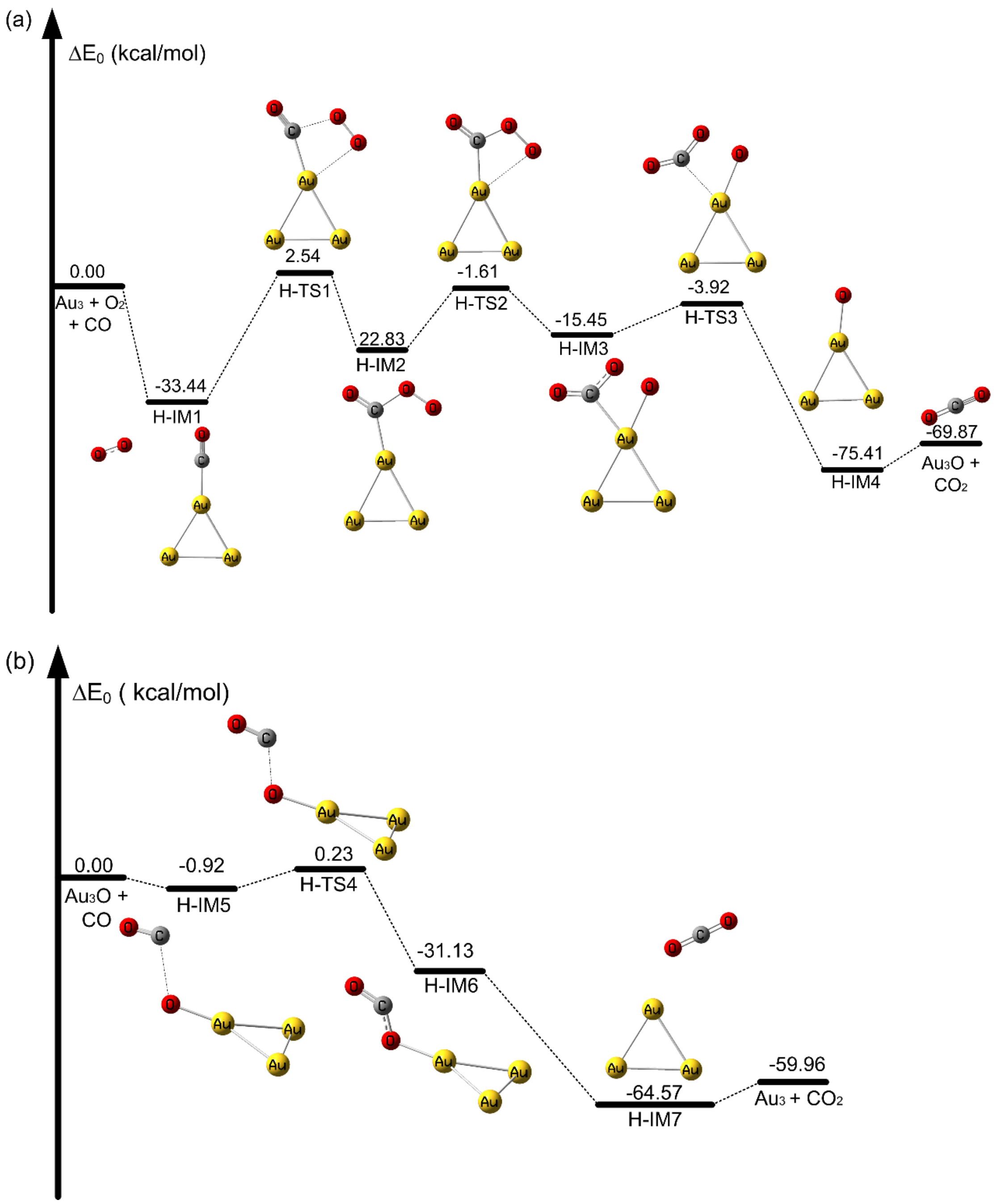

Figure S7. Potential energy profile of CO oxidation on Au_3 atoms at the CAM-B3LYP/aug-cc-pVDZ-PP level. (a) Oxidation of CO on the Au_3 cluster and (b) oxidation of CO on the OAu_3 cluster (in kcal/mol).

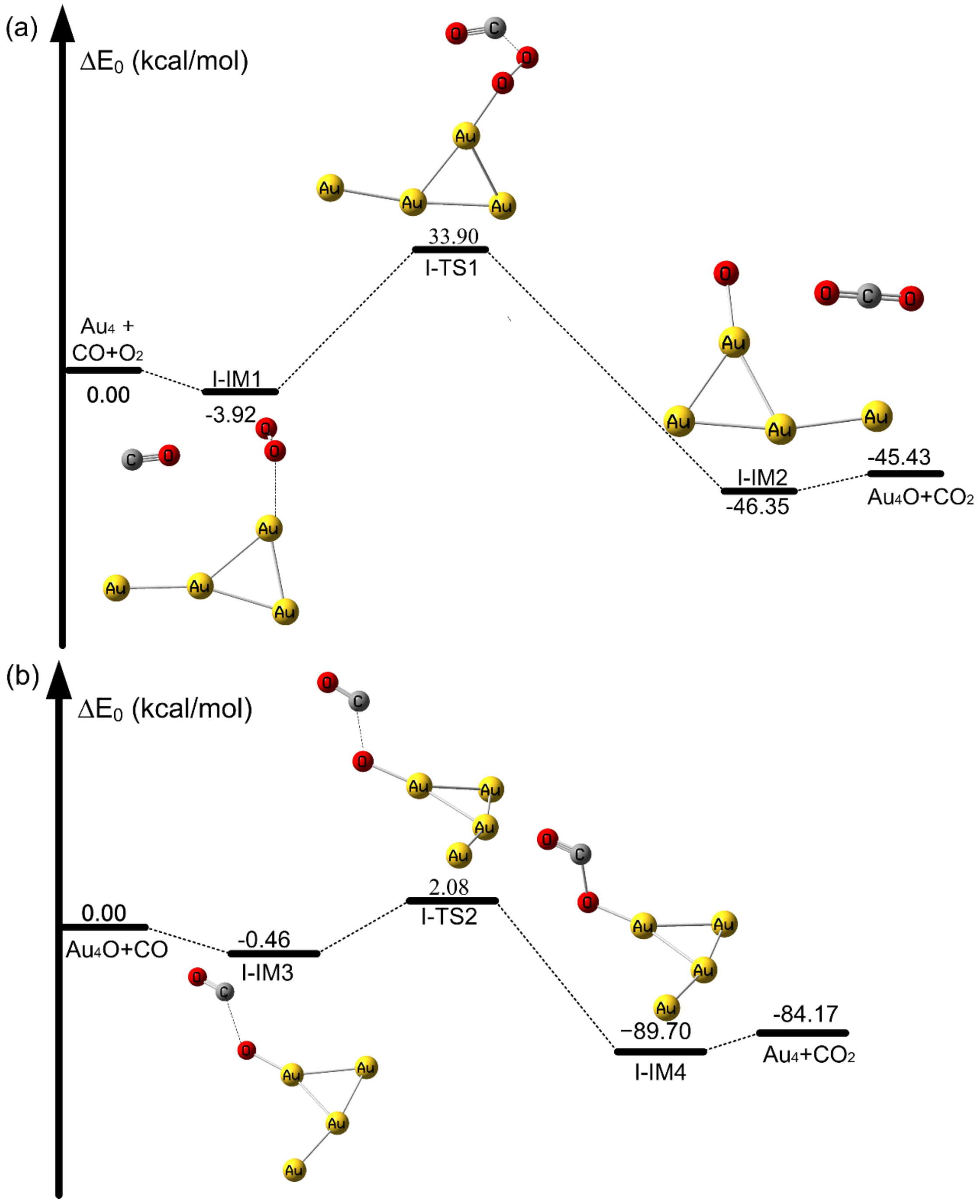

Figure S8. Potential energy profile of CO oxidation on Au_4 atoms at the CAM-B3LYP/aug-cc-pVDZ-PP level. (a) Oxidation of CO on the Au_4 cluster and (b) oxidation of CO on the OAu_4 cluster (in kcal/mol).

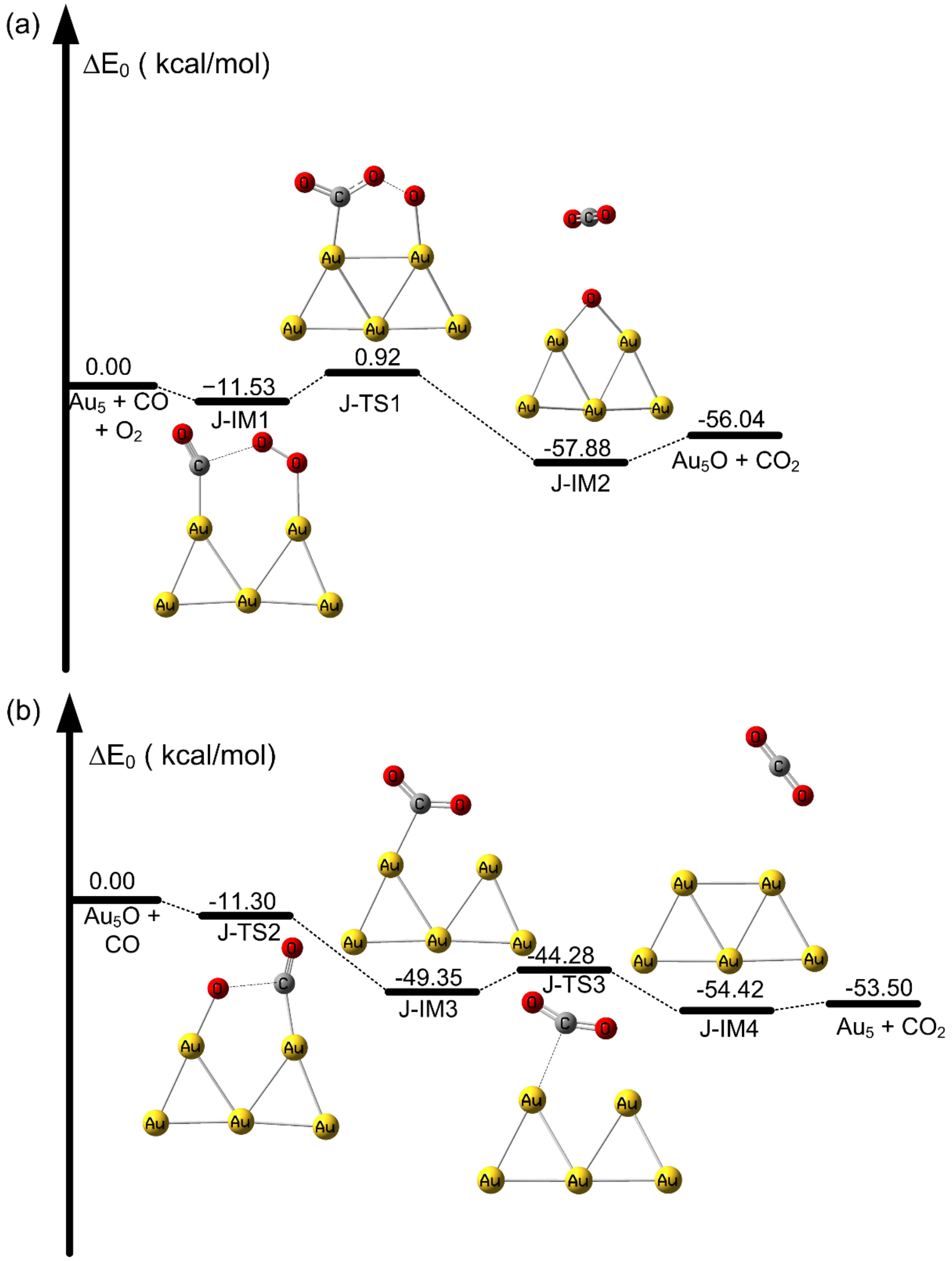

Figure S9. Potential energy profile of CO oxidation on Au_5 atoms at the CAM-B3LYP/aug-cc-pVDZ-PP level. (a) Oxidation of CO on the Au_5 cluster and (b) oxidation of CO on the OAu_5 cluster (in kcal/mol).

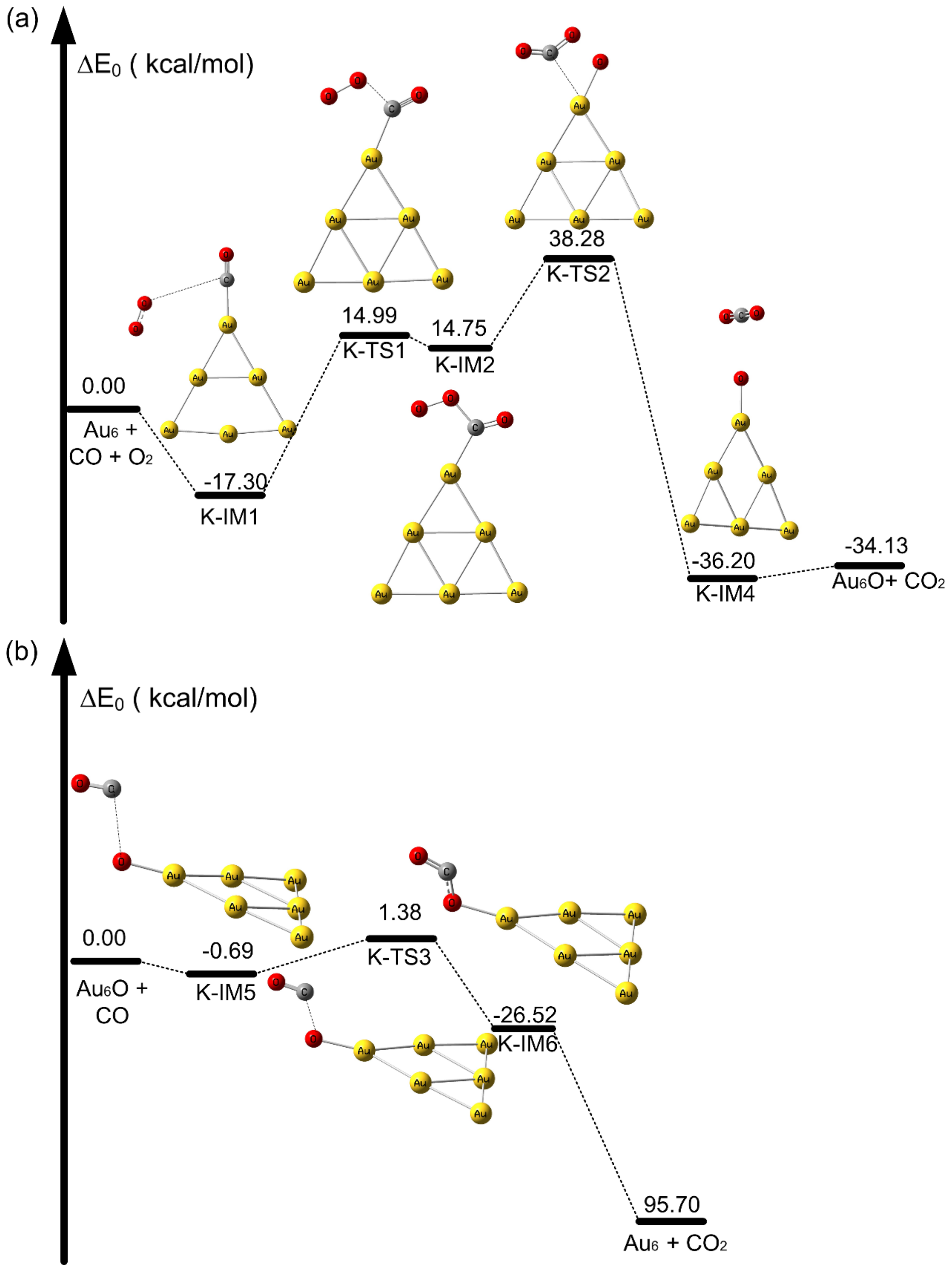

Figure S10. Potential energy profile of CO oxidation on Au_6 atoms at the CAM-B3LYP/aug-cc-pVDZ-PP level. (a) Oxidation of CO on the Au_6 cluster and (b) oxidation of CO on the OAu_6 cluster (in kcal/mol).

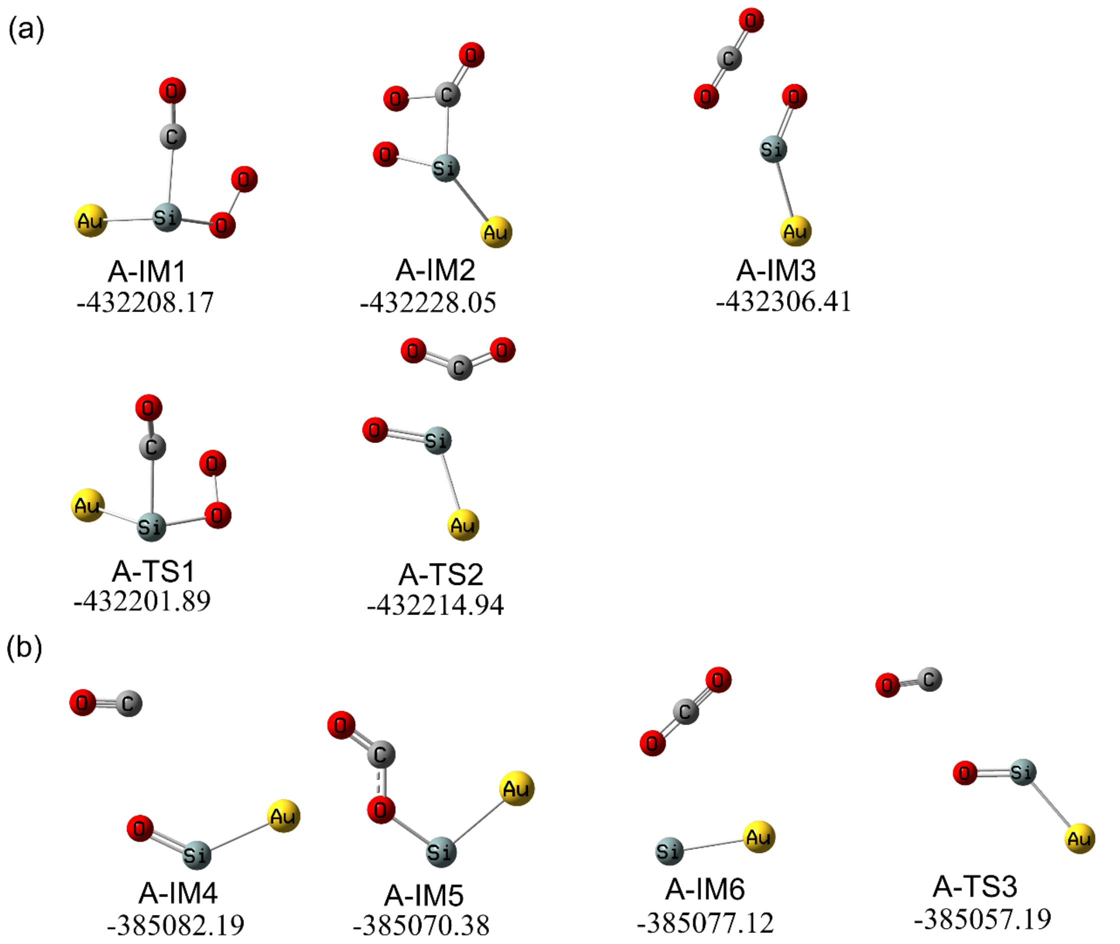

Figure S11. The structure and energy of various substances in the oxidation pathway for CO on AuSi (in kcal/mol).

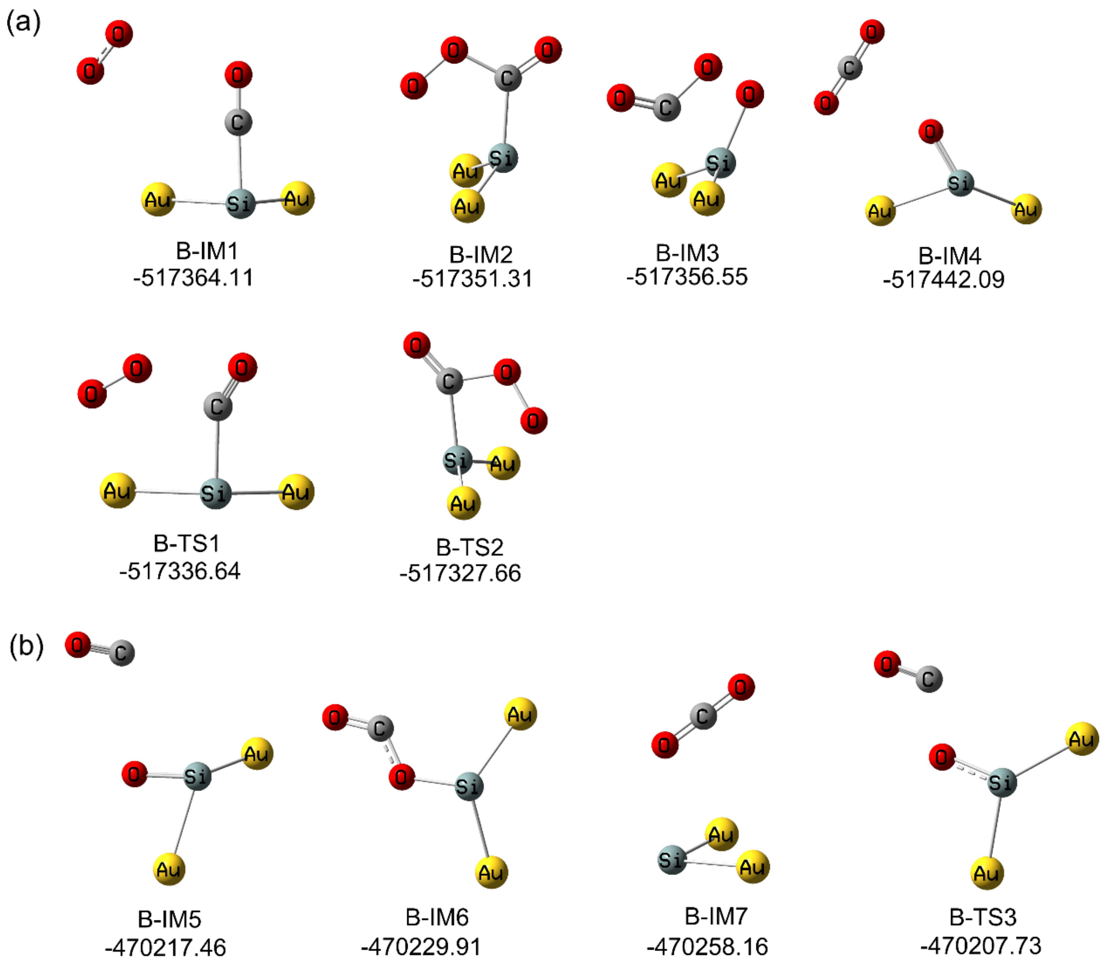

Figure S12. The structure and energy of various substances in the oxidation pathway for CO on Au_2Si (in kcal/mol).

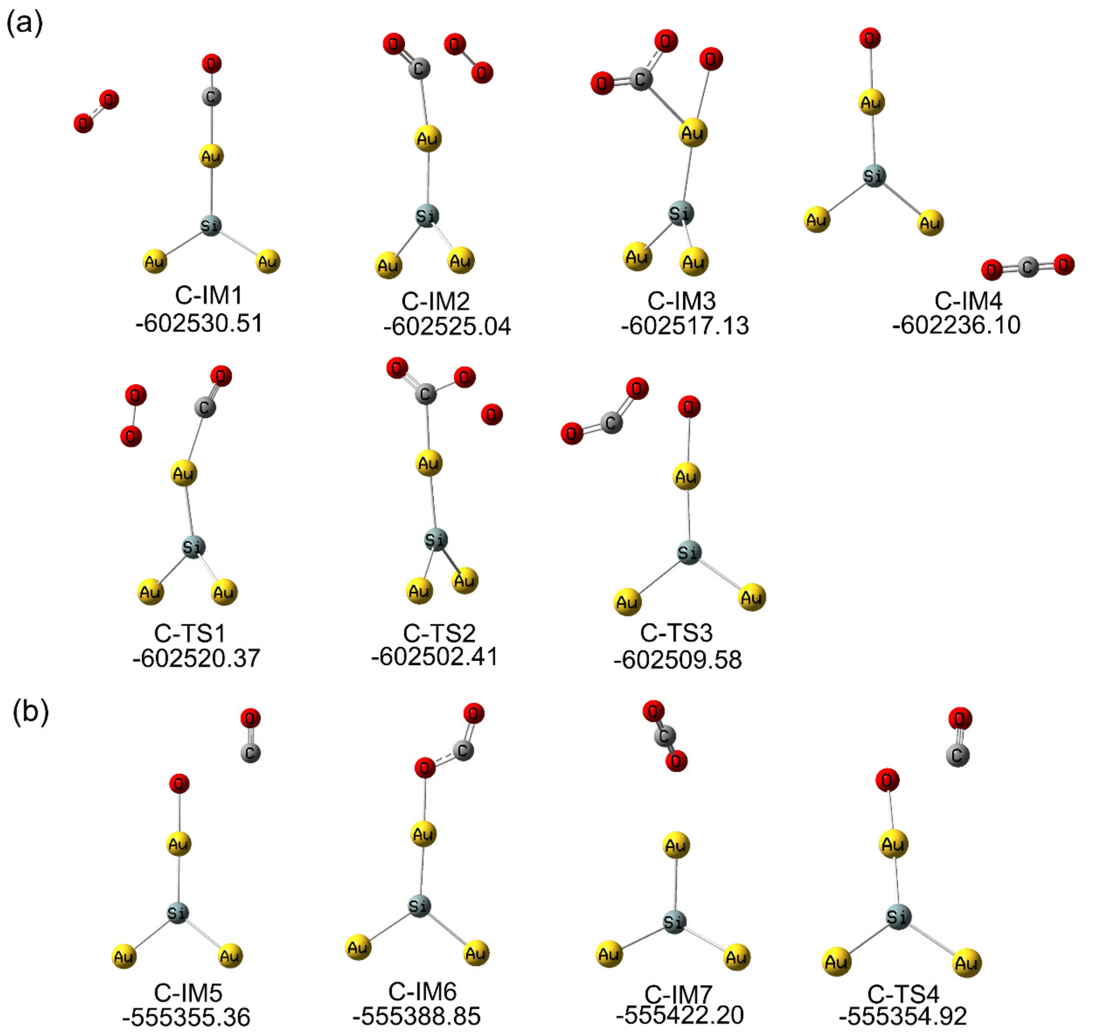

Figure S13. The structure and energy of various substances in the oxidation pathway for CO on Au_3Si (in kcal/mol).

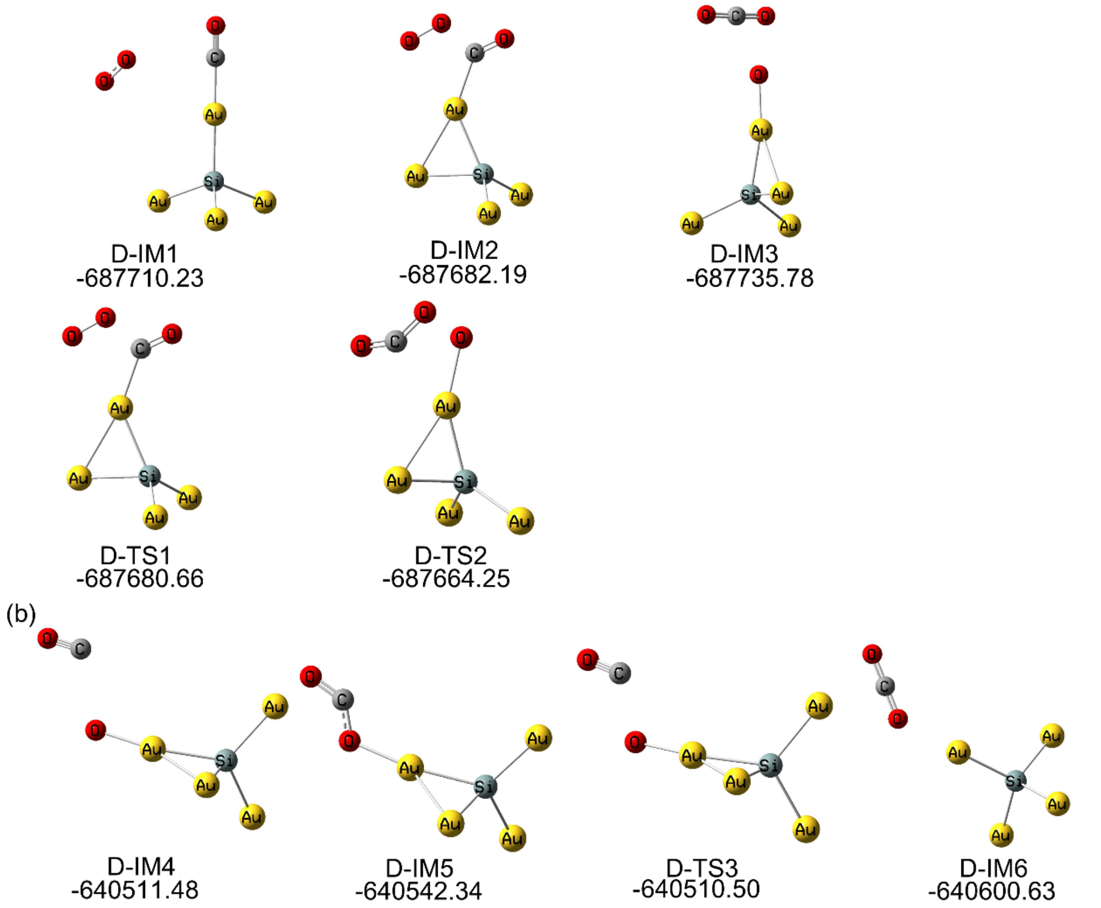

Figure S14. The structure and energy of various substances in the oxidation pathway for CO on Au_4Si (in kcal/mol).

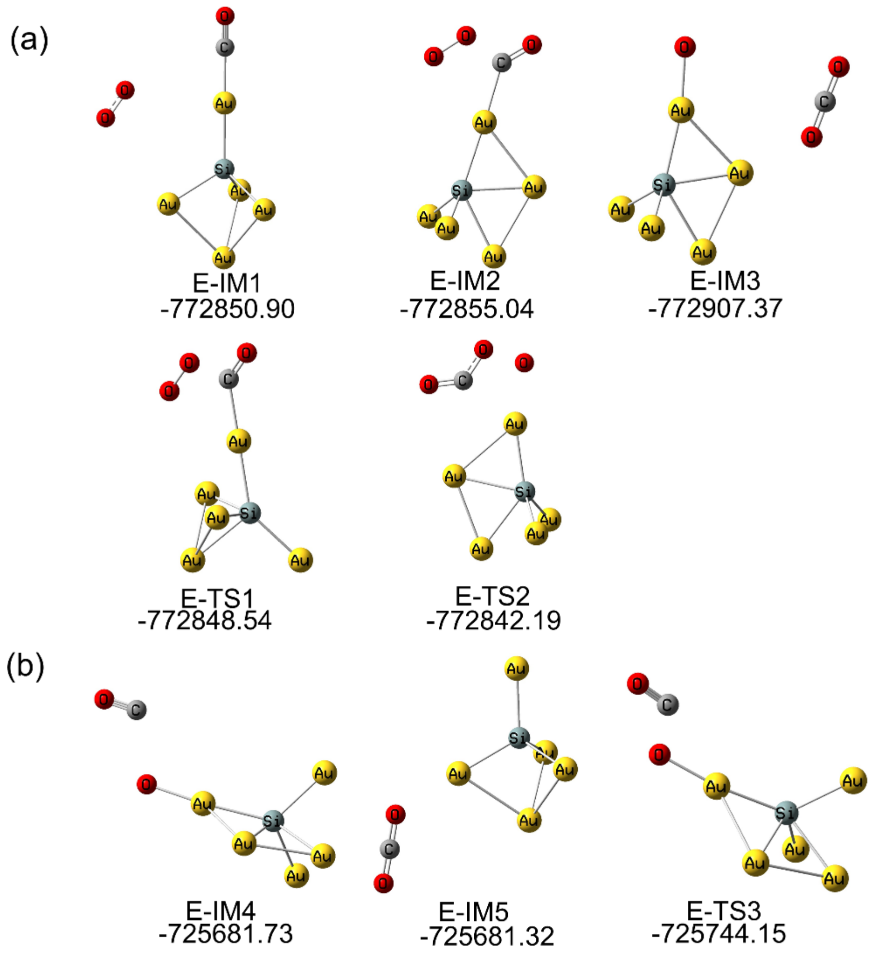

Figure S15. The structure and energy of various substances in the oxidation pathway for CO on Au_5Si (in kcal/mol).
